# Self-Controlled Learning: The Importance of Protecting Perceptions of Competence

**DOI:** 10.3389/fpsyg.2012.00458

**Published:** 2012-11-02

**Authors:** Suzete Chiviacowsky, Gabriele Wulf, Rebecca Lewthwaite

**Affiliations:** ^1^Physical Education School, Federal University of PelotasPelotas, Brazil; ^2^Department of Kinesiology and Nutrition Sciences, University of NevadaLas Vegas, NV, USA; ^3^Rancho Los Amigos National Rehabilitation Center, University of Southern CaliforniaDowney, CA, USA

**Keywords:** motor learning, autonomy support, perceived competence, self-efficacy, feedback

## Abstract

Recent studies examining the role of self-controlled feedback have shown that learners ask for feedback after what they believe was a “good” rather than “poor” trial. Also, trials on which participants request feedback are often more accurate than those without feedback. The present study examined whether manipulating participants’ perception of “good” performance would have differential effects on learning. All participants practiced a coincident-anticipation timing task with a self-controlled feedback schedule during practice. Specifically, they were able to ask for feedback after 3 trials in each of three 10-trial practice blocks. While one group (Self-30) was told that an error of 30 ms or less would be considered good performance, another group (Self-4) was informed that an error of 4 ms or less would be considered a good trial. A third, self-control group (Self) did not receive any information about what constituted good performance. The results showed that participants of all groups asked for feedback primarily after relatively good trials. At the end of practice, both the Self-30 and Self groups demonstrated greater perceived competence and self-efficacy than the Self-4 group. The Self-30 and Self groups also performed with greater accuracy and less variability in retention and transfer (non-dominant hand) 1 day later. The present findings indicated that the typical learning benefits of self-controlled practice can be thwarted by depriving learners of the opportunity of experiencing competence through good performance. They add to the accumulating evidence of motivational influences on motor learning.

## Introduction

Converging evidence suggests that providing learners with a certain degree of self-control, or autonomy, benefits motor learning. Advantages of self-control relative to yoked control groups have been demonstrated for the provision of feedback (e.g., Janelle et al., 1997; Chiviacowsky and Wulf, 2002; Huet et al., 2009; Patterson and Carter, 2010), order of trials during multi-task practice (Keetch and Lee, 2007; Wu and Magill, 2011), model observation (Wulf et al., 2005), use of assistive devices (Wulf and Toole, 1999; Wulf et al., 2001; Hartman, 2007), amount of practice (Post et al., 2011), and task difficulty (Andrieux et al., 2012). Self-controlled practice has been shown to benefit motor learning not only in adults but also in children (e.g., Chiviacowsky et al., 2008), older adults (Alcântara et al., 2007), and people with disabilities (Chiviacowsky et al., 2012).

Providing participants with some freedom of choice has been shown to benefit performance and learning in other domains as well (Cordova and Lepper, 1996; Tafarodi et al., 1999). For example, adults working in a high task-autonomy condition, in which participants were given considerable freedom and independence in deciding how to carry out a task (Hacken and Oldham, 1976), found more creative solutions to administrative problems than in a low task-autonomy condition (Zhou, 1998). Children provided with a higher level of autonomy on an arithmetic task showed increases in intrinsic motivation, depth of engagement, perceived competence, and learning (Cordova and Lepper, 1996). Allowing individuals to exercise control over the environment may satisfy a basic psychological need (White, 1959; Deci and Ryan, 2000, 2008), and/or biological necessity (Leotti et al., 2010; Leotti and Delgado, 2011). Studies with both humans (Tiger et al., 2006) and animals (Voss and Homzie, 1970; Catania, 1975; Catania and Sagvolden, 1980) have shown that both prefer an option leading to a choice than an option that does not, even if this option results in greater effort or work. These results suggest the existence of an inherent reward with the exercise of control. Choice, or the anticipation of the opportunity for choice, is associated with increased activity of brain regions directly involved in reward processing, and is greater than it is for anticipation of reward in the absence of choice (Leotti and Delgado, 2011).

Only a few studies have attempted to examine the underlying reasons for the benefits of self-controlled practice in motor learning. Some theoretical explanations have been proposed – borrowing from the verbal or cognitive learning domain where self-regulated learning has been more widely discussed – but they are relatively vague when applied to motor learning. For example, it has been suggested that more active involvement of the learner in the learning process promotes deeper processing of relevant information (Watkins, 1984; McCombs, 1989; Chen and Singer, 1992); that it encourages the use of self-regulation strategies (Kirschenbaum, 1984); or that giving the learner control over the practice conditions might be, in a general sense, more motivating (Bandura, 1993; Boekaerts, 1996). Motor learning researchers have largely adopted the explanations, although, empirical tests of these hypotheses still appear to be lacking.

One line of evidence points to the role of motivational factors associated with self-control as a possible explanation for the observed learning benefits. In a study by Chiviacowsky and Wulf (2002), participant interviews showed that (a) self-controlled learners asked for feedback predominantly after they believed they had a “good” trial, (b) errors on feedback trials were indeed smaller than those on no-feedback trials, and (c) yoked learners indicated that they would have preferred feedback after successful trials. These findings have since been replicated (e.g., Chiviacowsky et al., 2008; Patterson and Carter, 2010; Patterson et al., 2011). This suggests that the opportunity to request information after successful performance may play an important role in self-controlled learning. Moreover, follow-up studies have shown that providing learners with feedback after “good” relative to “poor” trials can enhance learning (e.g., Chiviacowsky and Wulf, 2007; Chiviacowsky et al., 2009; Saemi et al., 2011; Badami et al., 2012). It is possible that increased perceptions of competence and/or heightened self-efficacy – accruing from the opportunity to confirm successful movement outcomes, while “ignoring” less successful attempts – play a mediating role in this process. Higher levels of self-efficacy, defined as an individual’s belief regarding her or his ability to produce a desired result (Bandura, 1977), can lead to enhanced performance in various domains (Bandura, 1993; Feltz et al., 2008; Hutchinson et al., 2008). Similarly, the desire for competence is conceptualized as a drive to feel effective in interactions with the environment (White, 1959; Deci and Ryan, 1985, 2008; Conroy et al., 2007). A desire for competence is considered a basic psychological need, along with the needs for autonomy and social relatedness (i.e., the desire to feel connected to, and accepted by, others; Deci and Ryan, 2000). The satisfaction of all three psychological needs is believed essential for ongoing psychological growth and well-being – leading to more effective learning and maintained engagement with the task (Deci and Ryan, 2008; Sheldon and Filak, 2008).

The purpose of the present study was to examine more directly if the opportunity to select feedback after good trials – with potential concomitant effects on perceived competence and self-efficacy – is critical for the typically seen benefits of self-controlled feedback. If this were the case, depriving performers of the opportunity to feel competent under self-controlled practice conditions would be expected to have detrimental effects on learning. We sought to manipulate participants’ perceived competence in the present study by providing them with a standard or reference for “good performance.” We assumed that a high performance criterion that was difficult to meet would result in few ostensibly successful trials and thus would degrade perceptions of competence, self-efficacy and, consequently, motor learning. We also asked whether a criterion that was relatively easy to reach or surpass would provide learners with a boost in perceived competence and self-efficacy that could in turn enhance learning. Three groups of participants practiced a novel anticipation timing task and were able to request feedback about their temporal accuracy after 3 trials in each of three 10-trial blocks. Two of the groups were provided different criteria regarding what would be considered good performance (i.e., errors of 30 vs. 4 ms or less, respectively), thereby influencing their opportunities to conclude that they had a successful trial. The third, a self-control only group was not given a criterion for success. Questionnaires completed by participants at the end of the practice phase were used to determine participants’ perceived competence, self-efficacy, and task interest and enjoyment and their possible role in mediating learning. In particular, we were interested in whether self-efficacy ratings would predict learning (i.e., retention and transfer performance; Stevens et al., 2012).

## Materials and Methods

### Participants

Fifty-one university students (27 males, 24 females), all right-handed, with a mean age of 21.8 years (SD: 3.36) participated in this experiment. Participants were not aware of the specific purpose of the study and had no prior experience with the experimental task. All participants gave their informed consent. The study was approved by the university’s institutional review board.

### Apparatus and task

A Bassin anticipation timer (Model 35575, Lafayette Instruments, Lafayette, IN, USA) was used to measure temporal accuracy in anticipatory timing. The apparatus consisted of a 228 cm long track with 48 light-emitting diodes (LEDs) on its surface. The sequential illumination of the LEDs created the perception of a luminous red light moving down the runway. To increase the difficulty of the task, a barrier was placed over the trackway so that the 15 lights before the last one (target light) were obscured. Thus, participants had to anticipate the illumination of the target light. The task consisted of pressing a hand-held switch with the thumb of the preferred hand coincident with the illumination of the (last) target light. The task was performed from a seated position while facing the apparatus. A yellow warning light, used to cue the initiation of each trial, was set to illuminate for a variable period of time (2–5 s). The (perceived) running light moved at a constant speed of 20 mph.

### Procedure

Participants were randomly assigned to one of three groups (Self-30, Self-4, Self). They were told that the task consisted of pressing the hand-held switch, with the thumb of their preferred hand, coincidently with the illumination of the (last) target light, and that pressing the switch coincident with the target light illumination would correspond to a 0 ms error. In addition, participants in the Self-30 group were informed that an error of 30 ms or less would be considered good performance, whereas participants in the Self-4 group were told that an error of 4 ms or less constituted good performance. The third, self-control only group (Self) did not receive any performance standard and served as a control group. All participants practiced the coincident-anticipation timing task with a self-controlled feedback schedule. Specifically, they were able to ask for feedback after 3 trials in each 10-trial practice block, and received feedback directly after each request. Feedback consisted of the number of milliseconds the switch was pressed before or after the illumination of the target light, including error direction (e.g., −21 ms). They were also informed that they would have to perform the task without feedback on the following day. During the practice phase, all participants performed 30 trials. Retention and transfer tests were performed 1 day after the practice phase, each consisting of 10 trials without feedback. The transfer test, in which the participants were asked to use their non-dominant hand to press the switch, was performed 5 min after the retention test.

At the end of the practice phase, all participants completed a self-efficacy questionnaire and the perceived competence and task interest/enjoyment subscales of an adapted version of the Intrinsic Motivation Inventory (IMI; McAuley et al., 1989). On the self-efficacy questionnaire, participants were asked to rate how confident they were, on a scale from 1 (“not at all”) to 10 (“very”), that their errors would be smaller than 50 and 30 ms, respectively, the next day. The perceived competence subscale consisted of six statements (“I think I am pretty good at this task,” “I think I did pretty well at this task, compared to other participants,” “After practicing this task for a while, I felt pretty competent,” “I am satisfied with my performance at this task,” “I was pretty skilled at this task,” and “This was a task that I couldn’t do very well”). The task interest/enjoyment subscale of the IMI included seven statements (“I enjoyed doing this task very much,” “This task was fun to do,” “I thought this was a boring task,” “This task did not hold my attention at all,” “I would describe this task as very interesting,” “I thought this task was quite enjoyable,” and “While I was doing this activity, I was thinking about how much I enjoyed it”). Participants rated their perceived competence and interest/enjoyment on 7-point scales, ranging from 1 (“strongly disagree”) to 7 (“strongly agree”). Cronbach’s alpha coefficient indicated excellent consistency for perceived competence (0.91) and good internal consistency for interest/enjoyment (0.82). Finally, participants were asked when or why they asked for feedback during practice (as in Chiviacowsky and Wulf, 2002).

### Data analysis

Absolute error (AE), variable error (VE), and constant error (CE) were averaged across blocks of 5 (practice) and 10 (retention and transfer) trials. The practice data were analyzed in a 3 (groups) × 6 (blocks of 5 trials) analysis of variance (ANOVA) with repeated measures on the last factor. Separate one-way ANOVAs were used for the retention and transfer test data. To determine whether participants tended to ask for feedback predominantly after good trials during practice, the average AEs of trials with and without feedback were calculated and analyzed in a 3 (groups) × 2 (trial type: feedback, no-feedback) × 3 (blocks of 10 trials) ANOVA with repeated measures on the last two factors. Perceived competence and task interest/enjoyment scores were averaged across items and analyzed in one-way ANOVAs. Self-efficacy ratings were averaged across the two task difficulty levels (50 and 30 ms) and analyzed in a one-way ANOVA. Tukey’s *post hoc* test was used for follow-up analyses. Finally, we conducted linear regression analyses to determine whether self-efficacy predicted performance on the retention and transfer tests.

## Results

### Temporal accuracy

#### Practice

**Figure 1:**
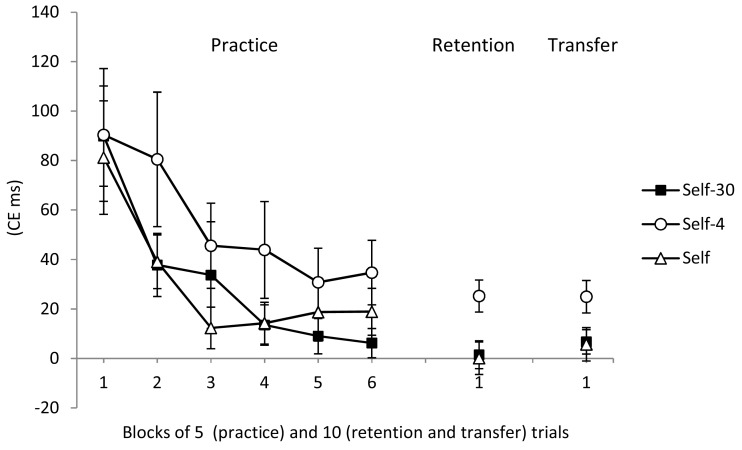
**Constant error during practice, retention, and transfer for the Self-30, Self, and Self-4 groups**. Error bars indicate standard errors.

All participants reduced their CEs during the practice phase (see Figure 1, left). The main effect of block was significant, *F*(5, 240) = 12.11, *p* < 0.01, η^2^ = 0.20, while the main effect of group, *F*(1, 48) = 1.54*, p* > 0.05, and the Group × Block interaction, *F*(10, 240) < 1, were not significant.

**Figure 2:**
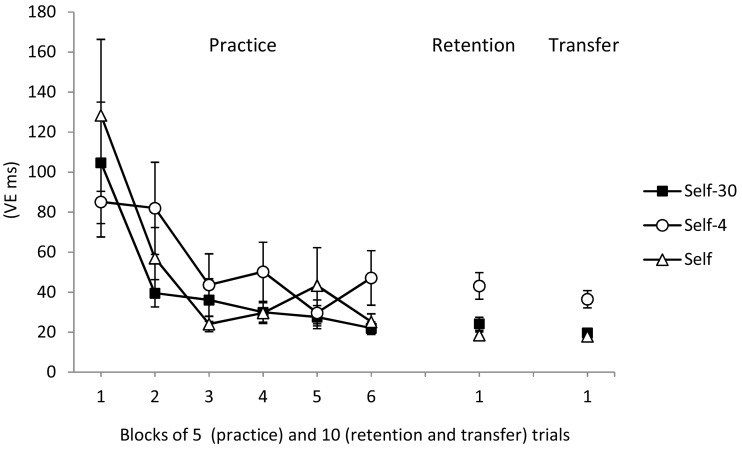
**Variable error during practice, retention, and transfer for the Self-30, Self, and Self-4 groups**. Error bars indicate standard errors.

All groups also reduced their temporal variability (VE) across practice (see Figure 2, left). The main effect of block was significant, *F*(5, 240) = 10.67, *p* < 0.01, η^2^ = 0.18. The main effect of group, *F*(1, 48) < 1, and the Group × Block interaction, *F*(10, 240) < 1, were not significant.

**Figure 3:**
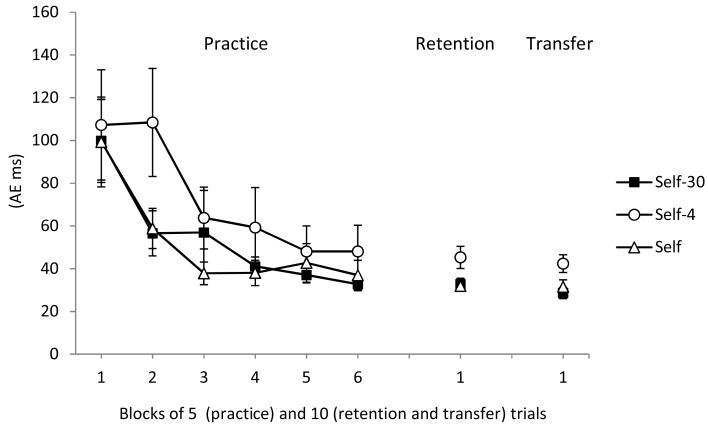
**Absolute error during practice, retention, and transfer for the Self-30, Self, and Self-4 groups**. Error bars indicate standard errors.

Finally, AEs decreased across the practice phase (see Figure 3, left). The main effect of block was significant, *F*(5, 240) = 11.76, *p* < 0.01, η^2^ = 0.19. The main effect of group, *F*(1, 48) = 1.71*, p* > 0.05, and the Group × Block interaction, *F*(10, 240) < 1, were not significant.

#### Retention

On the no-feedback retention test, the Self-30 and Self groups outperformed the Self-4 group (see Figure 1, middle). The group main effect was significant for CE, *F*(2, 51) = 5.14, *p* < 0.01, η^2^ = 0.17. *Post hoc* tests confirmed that both the Self-30 and Self groups had smaller CEs than the Self-4 group, *p*s < 0.05. There was no difference between the Self-30 and Self groups, *p* > 0.05.

VEs were also smaller in both the Self-30 and Self groups compared with the Self-4 group (see Figure 2, middle). The group main effect was significant, *F*(2, 51) = 8.58, *p* < 0.01, η^2^ = 0.26. *Post hoc* tests confirmed that both the Self-30 and Self groups had smaller VEs than the Self-4 group, *p*s < 0.01. There was no difference between the Self-30 and Self groups, *p* > 0.05.

The Self-30 and Self groups also had smaller AEs than the Self-4 group (see Figure 3, middle). The group main effect was significant, *F*(2, 51) = 4.21, *p* < 0.05, η^2^ = 0.14. *Post hoc* tests confirmed the differences between the Self-30 and Self groups and the Self-4 group, *p*s < 0.05. There was no difference between the Self-30 and Self groups, *p* > 0.05.

#### Transfer

On the no-feedback transfer test, during which participants used their non-dominant hand, all groups had similar CEs (Figure 1, right). The Group main effect was not significant, *F*(2, 51) = 3.12, *p* > 0.05.

However, both the Self-30 and Self groups had smaller VEs than the Self-4 group (Figure 2, right). The Group main effect was significant, *F*(2, 51) = 11.09, *p* < 0.01, η^2^ = 0.31. *Post hoc* tests confirmed that the Self-30 and Self groups were significantly less variable than the Self-4 group, *p*s < 0.01, but did not differ from each other, *p* > 0.05.

The Self-30 and Self groups also had smaller AEs than the Self-4 group (Figure 3, right). The Group main effect was significant, *F*(2, 51) = 4.97, *p* < 0.05, η^2^ = 0.17. *Post hoc* tests indicated that the Self-30 and Self groups differed significantly from the Self-4 group, *ps* < 0.05, but not from each other, *p* > 0.05.

### Feedback versus no-feedback trials

At the end of practice, participants answered the question when or why they requested feedback (see Table 1). The majority (28 of 51, or 54.9%) of the participants reported that they asked for feedback mostly after they thought they had a good trial. Six participants (11.9%) indicated that they requested feedback after a supposedly poor trial. Relatively few participants checked the other options.

**Table 1:** **Responses to the question, “When/why did you ask for feedback?”**

	*N*	%
**SELF-30**		
Mostly after you thought you had a good trial	10	58.7
Mostly after you thought you had a bad trial	1	5.9
After good or bad trials equally	4	23.6
Randomly	0	
None of the previous ones	2	11.8
**SELF**		
Mostly after you thought you had a good trial	10	58.7
Mostly after you thought you had a bad trial	2	11.8
After good or bad trials equally	2	11.8
Randomly	2	11.8
None of the previous ones	1	5.9
**SELF-4**		
Mostly after you thought you had a good trial	8	47.1
Mostly after you thought you had a bad trial	3	17.6
After good or bad trials equally	3	17.6
Randomly	2	11.8
None of the previous ones	1	5.9

To determine whether participants actually requested feedback more after relatively successful trials, we calculated AEs for feedback and no-feedback trials during initial, middle, and final practice blocks. AEs were significantly smaller on trials for which feedback had been requested (49.6 ms) than on those for which it was not requested (63.5 ms). The main effects of trial type, *F*(1, 96) = 11.54, *p* < 0.01, η^2^ = 0.19, and block, *F*(2, 96) = 13.63, *p* < 0.01, η^2^ = 0.22, were significant. There were no differences between groups, *F*(2, 48) = 2.46, *p* > 0.05, and no interaction of group, blocks, and trial type, *F*(4, 96) < 1. Thus, participants in all groups preferred and asked for feedback predominantly after more accurate trials. On feedback trials, the Self-30 group had an error of 30 ms or less (indicating to them that the trial was “good”) on 53% of the trials, whereas the Self-4 group had an error of 4 ms or less on 6% on those trials (The self-30 group had an error of 4 ms or less on 9% of the trials, and the Self-4 group had an error of 30 ms or less on 43% of the trials).

### Perceived competence

The Self-30 (4.38) had the highest perceived competence ratings (on a scale from 1 to 7), followed by the Self (4.09) and Self-4 groups (3.16). The Group effect was significant, *F*(2, 48) = 3.54, *p* < 0.05, η^2^ = 0.12. *Post hoc* tests indicated that the Self-30 group rated their perceived competence significantly higher than the Self-4 group, *p* < 0.01. The Self group did not differ from either group, *p*s > 0.05.

### Self-efficacy

At the end of practice, participants rated on a scale from 1 to 10 how confident they were that they would be able to produce errors of less than 50 and 30 ms, respectively, on the next day. Self-efficacy ratings for these two task difficulty levels were averaged to yield a single self-efficacy score. Both the Self-30 (7.65) and the Self (7.38) group participants showed greater self-efficacy than Self-4 group (5.74) participants. The Group effect was significant, *F*(2, 48) = 4.70, *p* < 0.05, η^2^ = 0.16. *Post hoc* tests indicated that both the Self-30 and Self groups differed significantly from the Self-4 group, *p*s < 0.05. The Self-30 and Self groups did not differ from each other, *p* > 0.05.

Linear regression analyses were conducted to determine if self-efficacy after the practice phase was a significant predictor of learning. Even though self-efficacy did not predict retention performance, *F*(1, 49) = 2.96, *p* > 0.05, R = −0.24, it was a significant predictor of transfer test performance, *F*(1, 49) = 19.79, *p* < 0.01, R = −0.54, explaining 27.3% of the variance.

### Task interest/enjoyment

Different groups also experienced different levels of task interest and enjoyment. The Group effect was significant, *F*(2, 48) = 5.66, *p* < 0.01, η^2^ = 0.19. *Post hoc* tests indicated that the Self-30 group (6.31) had higher ratings than both the Self (5.40) and Self-4 (5.58) groups, *p*s < 0.05. The Self and Self-4 group did not differ from each other, *p* > 0.05.

## Discussion

The purpose of the present study was to shed some light on the reasons underlying the benefits of self-controlled feedback. In previous studies (e.g., Chiviacowsky and Wulf, 2002; Patterson and Carter, 2010), self-control learners preferred and chose feedback mostly after successful trials. Therefore, we asked whether this possibility might result in heightened perceptions of competence and self-efficacy that could contribute to the observed learning advantages in self-controlled conditions. We succeeded in manipulating learners’ perceptions of competence and related self-efficacy through information about what was considered successful performance. Relative to the Self-4 group, the other groups experienced higher self-efficacy (Self-30 and Self groups) and competence (Self-30 group). Importantly, learning (i.e., retention and transfer performance) was more effective in these two groups compared with Self-4 group participants who experienced lower perceived competence and self-efficacy. Interestingly, the Self-30 group that received both the opportunity for self-controlled feedback and the criterion-defined experience of successful performance on a majority of their feedback trials, also reported heightened task interest and enjoyment relative to both the Self and Self-4 groups. However, this affective augmentation in intrinsic task motivation was not associated with more effective motor learning in the present study.

Our findings confirm those of previous studies (e.g., Chiviacowsky and Wulf, 2002, 2005; Chiviacowsky et al., 2008; Patterson and Carter, 2010; Patterson et al., 2011) in that participants generally preferred and asked for feedback mainly after successful trials. This preference may reflect the expression of a basic psychological need for competence or participants’ insights that their learning is better when success or a correlate such as self-efficacy is experienced. Yet, while opportunities for self-controlled feedback were available for all groups, this self-control was not met with the validation of good performance for those in the Self-4 condition, in which only 6% of the practice trials were considered “good” according to the provided criterion. The lack of positive feedback or receipt of essentially negative performance information resulted in reduced perceptions of competence and self-efficacy, degrading learning. This finding of impaired learning with indications of poor performance is in line with the findings of several recent studies which demonstrated that feedback provided after “poor” trials is not as effective for learning as feedback provided after “good” trials (e.g., Chiviacowsky and Wulf, 2007; Saemi et al., 2012) and that negative normative feedback degrades learning relative to positive social-comparative feedback (Lewthwaite and Wulf, 2010; Wulf et al., 2010, 2012). Interestingly, in one of those studies, which included a control group with veridical but no social-comparative information (Lewthwaite and Wulf, 2010), negative feedback also hindered learning relative to the control condition. This was not the case in the present study in which self-control without competence augmentation served as a control condition.

The similarity of both the perceived competence/self-efficacy and learning findings in the Self and Self-30 groups is notable. It may suggest that satisfaction of more than one psychological need is superfluous for learning (but see Sheldon and Filak, 2008, for some evidence of an additive effect) or that autonomy support is sufficient by itself to boost both perceived competence/self-efficacy and learning (Tafarodi et al., 1999). The fact that the Self-4 group, despite having the same self-controlled feedback opportunities as the other two groups, did not experience the same psychological and learning benefits appears to indicate that when one need (competence feedback suggesting relatively poor performance) is thwarted, or counteracts the other’s (autonomy) effect, the benefits of one (e.g., autonomy) are reduced. Alternatively, the Self group findings suggest that enhancements to perceived competence and self-efficacy may be effects of autonomy support. In the present study, in which self-controlled conditions meant the receipt of relatively positive feedback, we cannot decouple autonomy from competence information – one nearly automatically confirms the other when participants are choosing better performance trials on which to receive feedback. However, self-control effects have been found in conditions with less obvious conveyance of perceived competence and self-efficacy, such as when fewer rather than more practice trials are chosen (Post et al., 2011), under conditions of chance in which illusions of control are generated (e.g., Langer, 1975), or when choice involves incidental or trivial options that would have no ostensible impact on perceived competence or self-efficacy (Cordova and Lepper, 1996; Tafarodi et al., 1999). Tafarodi and colleagues found confidence effects for experimental options that involved choosing among names to be used in reading comprehension assessments.

The results highlight the role of motivational influences on motor learning. From an information-processing perspective, no learning differences among groups would have been expected, as all groups experienced the same active engagement in the learning process and had the same opportunity to choose feedback. In pursuit of the motivational circumstances that optimize motor learning (Lewthwaite and Wulf, 2012), several related but distinct psychological constructs have been directly or indirectly studied, including perceived competence, self-efficacy, perceived choice, intrinsic motivation or task interest and enjoyment, and positive and negative affect. These cognitive and affective motivational variables have been influenced by motivational conditions, including autonomy support (i.e., learner control of some practice conditions) and social-comparative and other forms of augmented instructions or feedback. Future research can provide more definitive evidence of the conditions and constructs, as well as the neurophysiological (e.g., dopaminergic; Jay, 2003) or neural activation effects (Leotti et al., 2010; Leotti and Delgado, 2011) associated with particular practice conditions or related cognitions or affective experiences. To do so will involve the capacity to measure distinct, preferably theoretically derived, constructs, as well as the experimental power to detect differences among them. For example, perceived competence and self-efficacy have theoretical differences but share some common conceptual ground in perceived ability and associated affective consequences generated by these perceptions (Bandura, 1997). Yet, they diverge in a number of potentially important ways for skill acquisition. It may be the cognitive distinctions or affective similarities that contribute to their motivational impacts on motor learning.

## Conflict of Interest Statement

The authors declare that the research was conducted in the absence of any commercial or financial relationships that could be construed as a potential conflict of interest.
